# Age-specific epidemic waves of influenza and respiratory syncytial virus in a subtropical city

**DOI:** 10.1038/srep10390

**Published:** 2015-05-18

**Authors:** Lin Yang, Kwok Hung Chan, Lorna K P Suen, King Pan Chan, Xiling Wang, Peihua Cao, Daihai He, J S Malik Peiris, Chit Ming Wong

**Affiliations:** 1 School of Nursing, The Hong Kong Polytechnic University; 2 Department of Microbiology, The University of Hong Kong; 3 School of Public Health, The University of Hong Kong; 4 School of Public Health, Fudan University; 5 Department of Applied Mathematics, The Hong Kong Polytechnic University.

## Abstract

Both influenza and respiratory syncytial virus (RSV) are active throughout the year in subtropical or tropical regions, but few studies have reported on age-specific seasonal patterns of these viruses. We examined the age-specific epidemic curves of laboratory-confirmed cases of influenza A (subtyped into seasonal A(H1N1), A(H3N2), and pandemic virus A(H1N1)pdm09), influenza B and respiratory syncytial virus (RSV), in subtropical city Hong Kong from 2004 to 2013. We found that different types and subtypes of influenza showed similar two-peak patterns across age groups, with one peak in winter and another in spring/summer. Age differences were found in epidemic onset time and duration, but none could reach statistical significance (*p* > 0.05). Age synchrony was found in epidemic peak time for both cool and warm seasons. RSV showed less clear seasonal patterns and non-synchronized epidemic curves across age. In conclusion, age synchrony was found in influenza seasonal epidemics and the 2009 pandemic, but not in RSV. None of the age groups consistently appear as the driving force for seasonal epidemics of influenza and RSV in Hong Kong.

Studies on age-specific epidemic curves of respiratory virus infections could provide key evidence for the mechanism of virus transmission and the formulation of age-specific control measures^1^. Among all respiratory viruses, influenza and respiratory syncytial virus (RSV) attract the most attention because of the heavy disease burden directly or indirectly associated with these two viruses, especially among young children and the elderly^2^. In Hong Kong, influenza and RSV have overlapping seasonal epidemics, but different age distribution patterns^3^. Regional heterogeneity of influenza and RSV seasonality has also been well documented^4^,^5^. Both viruses have a clear winter peak in temperate regions, but in tropical and subtropical regions these two viruses can be active throughout the year^6^. Numerous attempts have been made to explore the seasonal factors that drive annual epidemics of influenza and RSV^7^,^8^. Proposed seasonal drivers include meteorological factors (temperature and humidity), host susceptibility and human behavior change, but the mechanism still remains largely unclear. Investigations of temporal synchrony in age-specific epidemic curves of respiratory viruses may add more hints to the search of seasonal drivers. However, the major obstacle to obtaining age-specific epidemic curves of respiratory viruses is the lack of virological surveillance data with linked demographical information in most countries. Even fewer such data are available in subtropical and tropical regions, where the year-round circulation of respiratory viruses further complicates the assessment of epidemic timing^5^. In this study, we utilized long-term age-specific surveillance data on influenza and RSV in subtropical Hong Kong, with the aim to explore the temporal relationship of virus activities across age under warm climates.

## Methods

### Data source

Virological data from 2004 to 2013 were obtained from the microbiology laboratory of Queen Mary Hospital (QMH). As part of the influenza surveillance network in Hong Kong, this laboratory serves a population of 1.2 million in Hong Kong Island (around 20% of the entire Hong Kong population). Our previous study has found that the surveillance data of this laboratory were highly correlated with those from the entire network^9^. Given the highly compacted population in this small area, influenza activity showed high geographical homogeneity in Hong Kong^10^. Hence we believe our data are representative of the entire Hong Kong population. Age information was available in more than 99.9% of the 120 571 specimens received for tests during the study period. Positive or negative cases with missing age were excluded from analysis. Nasopharyngeal swabs and aspirates were routinely collected from both inpatients and outpatients with influenza-like symptoms. During the whole study period, direct immunofluorescence (IF) tests were conducted to test influenza (type A or B) and RSV^11^. Of all 9952 specimens positive for influenza A, nearly 90% were subtyped into seasonal A(H3N2) and A(H1N1) by viral culture (Table 1). During the first wave of 2009 H1N1 pandemic, specimens were also tested for the pandemic virus A(H1N1)pdm09 by reverse-transcription polymerase chain reaction (RT-PCR). Age, gender, and date of specimen collection were obtained for each tested patient from his or her hospital records. The annual age-specific population size in Hong Kong Island was derived from the 2001, 2006, and 2011 censuses by linear interpolation.

### Data analysis

Weekly age-specific numbers and proportions of positive specimens (positive proportions) were aggregated from individual virological data for influenza A (subtypes A(H3N2), A(H1N1) and A(H1N1)pdm09), influenza B and RSV. Age groups of 0–4, 5–17, 18–64, and 65 + years were considered. There were 26 co-infection cases of influenza and RSV, which were separately counted for each infected virus. Around 10% of influenza A positive specimens were not subtyped. We then allocated the weekly numbers of these data into three subtypes according to the ratio of the annual total of each subtype in the corresponding year. We excluded the ratio of A(H1N1)pdm09 before the onset of the pandemic (26 April 2009) and separately calculated the annual ratio for the first 17 weeks of 2009 and the remaining 35 weeks.

The timing of the epidemic peaks of these viruses in different age groups was visualized by surface plots of weekly positive rates, which were defined as the weekly positive numbers divided by the age-specific population size. These rates were used to avoid the dominance of some age groups because of their large population size. The mean week of the epidemics (MWE) was also calculated to quantify the epidemic peak time, by influenza type(subtype) and season (warm/cool)^12^:


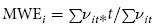


where 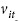 denotes the number of laboratory confirmed cases at week *t* of season 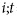 = 1,2,…, 50, -1,0 denotes the week number within the year, and the week number 51, 52 were replaced by -1, 0 in order to avoid the dominance of these large numbers in calculating the MWE of cool season. To compare the onset time and duration of influenza epidemics, we defined age-specific epidemics as a period of two or more consecutive weeks with at least 4% of the annual number of laboratory-confirmed cases for each age group for influenza A(H1N1), A(H3N2), A(H1N1)pdm09, and B^13^. The onset time of the epidemic was defined as the first week of the defined epidemic in each warm or cool season, regardless of how many epidemics were defined in that season. Considering two seasonal peaks of influenza, we calculated the MWE, onset time and duration of the epidemics in the warm season (weeks 19 to 50) and cool season (week 51 to week 18 of the following year), respectively.

We compared the duration of the epidemics across age groups for each virus using Poisson models. Because seasonal A(H1N1) was almost completely replaced by A(H1N1)pdm09 after 2009, only A(H1N1) data during January 2004 – December 2009 and A(H1N1)pdm09 after April 2009 were included in the analysis. All analyses were conducted in R package version 2.12.2 (R Foundation for Statistical Computing, Vienna, Austria). Ethical approval for this study was obtained from the Institutional Review Board of the University of Hong Kong/Hospital Authority Hong Kong West Cluster (UV11-264).

## Results

During the study period of 2004–2013, annual total number of specimens tested by QMH’s microbiology laboratory ranged from 6363 to 18906. In these specimens, overall positive proportions of influenza A, B and RSV were 18.9%, 21.0%, 12.7%, and 8.8% for the age groups of 0–4, 5–17, 18–64, and 65+, respectively. Influenza A was most frequently detected in these specimens, followed by RSV and influenza B (Table 1). For influenza, the pattern of age-specific positive proportions markedly varied between different types and subtypes. A(H3N2) was detected in all the age groups at similar frequency, whereas A(H1N1), A(H1N1)pdm09, and B showed higher positive proportions in schoolchildren aged 5–17 years than in the other age groups. Few people aged 65+ years were infected by A(H1N1), A(H1N1)pdm09, or influenza B. Children aged below 5 years had a high positive proportion for RSV (Table 1).

**Figure 1:**
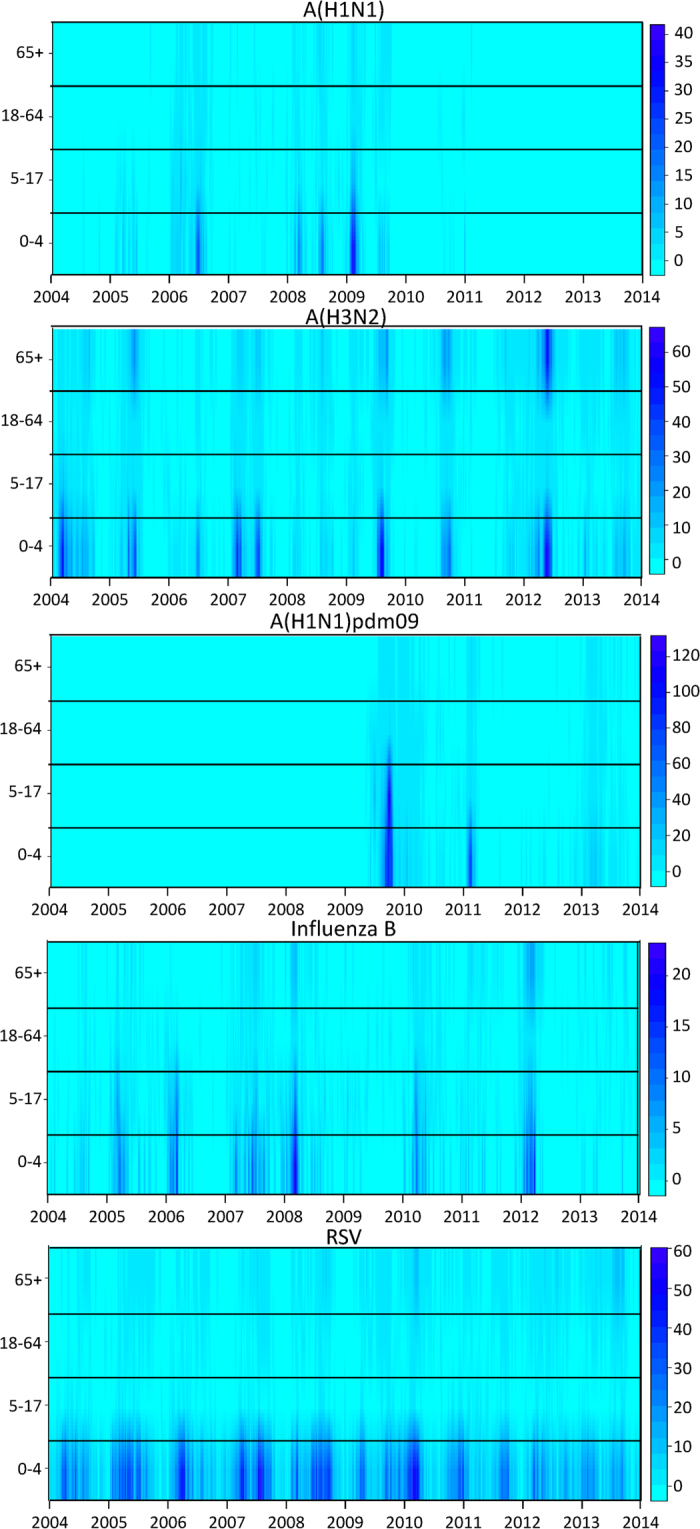
Surface plot of weekly positive rates (per 100 000 population) of laboratory confirmed influenza A(H1N1), A(H3N2), A(H1N1)pdm09, influenza B and respiratory syncytial virus (RSV) by age group. The color bar on the right of each panel represents the rates range from the lowest (light blue) to highest (dark blue).

The surface plots showed that all the types and subtypes of influenza viruses were featured with synchronized peaks across all the age groups in most of the study period (Fig. 1). All the age groups demonstrated similar seasonal patterns for influenza, with a few exceptions occasionally observed. For example, for A(H1N1) in 2005 and B in 2006, the peaks were observed only in children, but absent in adults and elderly persons. The first wave of the A(H1N1)pdm09 pandemic occurred around September 2009, and the second wave around February 2011. Many cases were confirmed in young children in both waves, but markedly fewer cases were found in schoolchildren in the second wave than in the first wave. Multiple peaks of RSV cases were observed among young children each year, and no clear patterns were observed in adults because of a limited number of cases. Highly synchronized virus activity across age groups was found in A(H1N1)pdm09.

**Figure 2:**
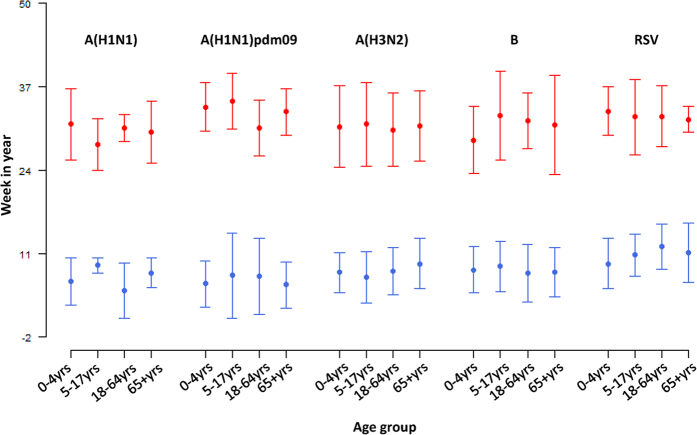
Mean week of the epidemics (MWE), 2004–2013. Influenza viruses A(H1N1), A(H1N1)pdm09, A(H3N2), B and respiratory syncytial virus (RSV) are separately plotted by age groups of 0-4, 5-17,18-64 and 65+. Red dots represent the mean weeks of the epidemics in warm season (week 19 to week 50), and blue dots represent those in cool season (week 51 to week 18 of next year). Week number ranges from -2 to 50, with negative indicating the weeks before that corresponding year (i.e. -2 represents week 50 of last year). The vertical bars indicate ± one standard error.

There was no consistent pattern observed for the epidemic peak time of influenza types and subtypes across age groups (Fig. 2). For A(H1N1), MWE showed different age patterns between warm and cool season, whereas for A(H1N1)pdm09 and influenza B, schoolchildren tended to slightly lag behind other age groups in both seasons. Compared with the other viruses, A(H3N2) appeared more synchronized across age, with small age differences found in MWE. For RSV, young children led the other age groups in winter peaks but lagged behind in summer.

**Figure 3:**
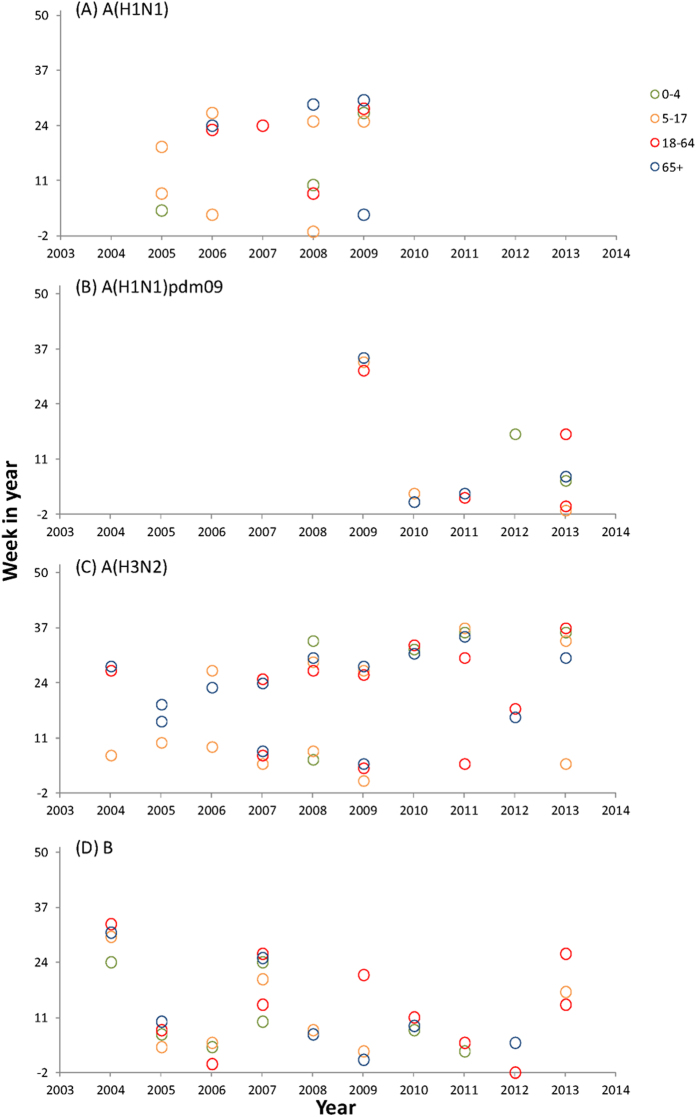
Onset time of influenza epidemics of (**A**) A(H1N1), (**B**) A(H1N1)pdm09, (**C**) A(H3N2) and (**D**) B, in cool or warm season of each year by age groups, 2004-2013. Week number ranges from -2 to 50, with negative indicating the weeks before that corresponding year (i.e. -2 represents week 50 of last year). Warm season is defined as week 19 to week 50, and cool season as week 51 to week 18 of next year. Threshold for epidemic is set at 4% of annual total age-virus-specific positive cases.

No consistent pattern was observed for the epidemic onset time of influenza types and subtypes across age groups (Fig. 3). For A(H1N1) and A(H3N2), schoolchildren led the seasonal epidemics more often than the other age groups, whereas the 0–4 age group had earlier onset time in the epidemics of influenza B . In cool season, the 5-17 age group led other age groups in 6 out of 8 seasons with the A(H3N2) epidemics defined, 2 out of 4 for the A(H1N1) epidemics, 1 out of 3 for the A(H1N1)pdm09 epidemics and 1 out of 8 for the influenza B epidemics. In warm season, the numbers of seasons led by the 5-17 age group was 0 out of 9, 3 out of 5, 0 out of 1 and 2 out of 4 for the epidemics of A(H3N2), A(H1N1), A(H1N1)pdm09 and influenza B, respectively. Compared with seasonal viruses, A(H1N1)pdm09 appeared more synchronized across age, with small gaps in the onset time between different age groups. The patterns became more irregular when the epidemic threshold was lowered from 4% to 2% (Supplementary Fig. 1).

Table 2 compares the age-specific epidemic durations across age groups in different types and subtypes of influenza. Not all the age groups reached the epidemic threshold every season under study. Among the types and subtypes of influenza, A(H3N2) had longer epidemics in all of the age groups. The elderly had shorter influenza epidemics than the other age groups. None of these age differences in terms of duration of the epidemics periods were found statistically significant (*p* > 0.05). Similar results were found when the epidemic threshold was changed from 4% to 2% (data not shown).

## Discussion

In this epidemiological study, we found influenza virus activity was synchronized across age groups, which suggests that seasonal influenza epidemics are probably mainly driven by the factors affecting all the age groups. Previous studies have suggested that meteorological factors, human behavior, antigenicity change of viruses and pre-existing immunity could all play important roles in determining the seasonal outbreaks of influenza. However, the mechanism is complicated and remains largely unknown^8^. Few studies have explored the mechanisms behind RSV seasonality, but we speculate that less synchronized RSV activity may be due to the fact that most severe cases were identified in young children and elderly persons, and mild cases of other age groups might not seek medical treatment who were unlikely captured by our surveillance system. Our findings further highlight the need to enhance the community surveillance for mild cases of respiratory infections with detailed demographic information. Incorporation of these data into the present surveillance at clinical settings could greatly improve our understanding of virus transmission and seasonal outbreaks. Viboud *et al.* has recently conducted a study to demonstrate that age-specific influenza-like illness (ILI) data from both medical claims and sentinel surveillance successfully captured the seasonal variations and epidemic timing of age-specific laboratory surveillance data^14^.

Two seasonal influenza A subtypes, A(H3N2) and A(H1N1), have been co-circulating in the human population since 1977^15^. Phylogenetic studies have shown that A(H1N1) has fewer antigenic variations and causes milder infections than A(H3N2)^16^,^17^. The difference of these two subtypes in age distribution has also been reported^18^. Similarly, in Hong Kong we found that A(H1N1) less likely infects the older population and most of the cases were found in children aged below 18 years, whereas A(H3N2) can attack all the age groups at similar frequency. This discrepancy in age distribution could be owing to fast antigenicity evolution and lack of pre-existing immunity against novel A(H3N2) in all the age groups. However, we did not find significant differences between these two subtypes in terms of seasonal pattern, epidemic timing and duration across age. Further studies with age- and subtype-specific virological data from more countries could help us better understand the potential disparity between different influenza A subtypes.

Previous studies have suggested that although the elderly have a high mortality and morbidity risk associated with influenza^19^,^20^, children could be the major driving force of influenza virus transmission in the human population^21^. For example, the effectiveness of school closure in containing community outbreaks has been demonstrated for both seasonal and pandemic influenza^22^,^23^. One study in Canada reported that schoolchildren aged 10–19 years led other age groups by one week in seasonal influenza peaks^24^. Proxies from children, such as emergency room visits for respiratory symptoms, provided timely alerts to influenza epidemics^25^. Although we found some evidence that schoolchildren were more likely to lead the A(H3N2) winter epidemics than other age groups, this was far from conclusive to support that children are the primary source of influenza transmission that drives seasonal epidemics in Hong Kong. Overall all the age groups demonstrated similar seasonal patterns, and there was no consistent age pattern observed in the epidemic peak or onset time, and the epidemic duration for each influenza type or subtype.

In line with the findings of a global study based on monthly data^26^, we also found that RSV exhibited a similar two-peak seasonal pattern as influenza, but with broad peaks in the weekly time resolution. RSV causes bronchiolitis in young children, and its associated disease burden in the elderly was under-recognized for a long time^2^. Our study found many RSV infections in the 0–4 and 65+ age groups but very few in the 5–17 and 18-64 age groups. Age-specific epidemic curves of RSV appeared more scattered and less synchronized across different age groups than those of influenza.

Our study has several limitations. First, most of our specimens were tested by IF, which has lower specificity and sensitivity than RT-PCR. For influenza A, IF provides satisfactory test results, with sensitivity and specificity of up to 96% and 99.6%, respectively, in children (with RT-PCR as the gold standard)^27^. However, IF is less sensitive to influenza B, and its performance in specimens collected from adults is suboptimal^28^. This discrepancy between age and virus type in IF test performance can have affected the temporal correlation of age-specific test-positive cases. Nevertheless, since this test has been consistently adopted for all the samples, we believe the temporal variation of positive IF results can still reliably reflect the true variation of influenza cases within each age-virus group. Second, our data were obtained from people who sought for medical treatment in two hospitals, thereby likely representing relatively more severe cases. Therefore our results may not reflect the relative timing of virus activity across different age groups in community outbreaks. To better understand the age pattern of all infected cases, ideally a random sample of people with influenza-like symptoms should be drawn from the community, but such a study can be hindered by enormous demands on financial cost and manpower. Nevertheless, age pattern of infected cases that required medical treatment still provides valuable information on age profile of different respiratory viruses and can thus facilitate the formulation of control measures specifically targeting on different age groups.

Our study for the first time analyzed the age-specific seasonal patterns of influenza and RSV in subtropical city Hong Kong. The large numbers of specimens taken from each age group allowed us to compare the influenza epidemic timing and duration across age groups. Our findings highlight the complexity in virus transmission under warm climates and also the need to enhance data collection of age information in routine virological surveillance in other subtropical and tropical regions.

## Author Contributions

L.Y., K.H.C., C.M.W. designed the study; K.H.C. collected the data; L.Y., K.P.C., X.W., P.C. cleaned the data; L.Y. conducted the data analysis; L.Y. and K.H.C. drafted the manuscript; L.K.P.S., D.H., J.S.M.P. and C.M.W. revised the manuscript.

## Additional Information

**How to cite this article**: Yang, L. *et al.* Age-specific epidemic waves of influenza and respiratory syncytial virus in a subtropical city. *Sci. Rep.* **5**, 10390; doi: 10.1038/srep10390 (2015).

## Supplementary Material

Supplementary Information

## Figures and Tables

**Table 1:** Total numbers and proportions (%) of specimens positive for respiratory viruses by age group, 2004–2013.

	**0–4yrs**		**5–17yrs**		**18–64 yrs**		**65+ yrs**		**No age**	**All ages**	
	**No.**	**(%)**^a^	**No.**	**(%)**^a^	**No.**	**(%)**^a^	**No.**	**(%)**^a^	**No.**	**No.**	**(%)**^a^
Total specimens	33151		12814		24313		50179		114	120571	
Influenza A	2559	(7.7)	1979	(15.4)	2453	(10.1)	2951	(5.9)	10	9952	(8.3)
A(H3N2)	1250	(3.8)	511	(4.0)	926	(3.8)	2358	(4.7)	5	5050	(4.2)
A(H1N1)	206	(0.6)	134	(1.0)	151	(0.6)	109	(0.2)	0	600	(0.5)
A(H1N1)pdm09	752	(2.3)	1095	(8.5)	1193	(4.9)	228	(0.5)	0	3268	(2.7)
Nonsubtyped	351	(1.1)	239	(1.9)	183	(0.8)	256	(0.5)	5	1034	(0.9)
Influenza B	371	(1.1)	533	(4.2)	272	(1.1)	331	(0.7)	2	1509	(1.3)
RSV	3322	(10.0)	177	(1.4)	364	(1.5)	1141	(2.3)	8	5012	(4.2)

^a^Age-specific positive proportions.

**Table 2:** Comparison of annual epidemic durations (in weeks) across age groups, 2004-2013. Zero indicates no epidemic was defined in some years.

	**0–4yrs**			**5–17yrs**			**18–64yrs**			**65+ yrs**		
**Virus**	**mean**	**(range)**	**p-value**^**#**^	**mean**	**(range)**	**p-value**^**#**^	**mean**	**(range)**	**p-value**^**#**^	**mean**	**(range)**	**p-value**^**#**^
A(H1N1)	3.8	(0,10)	Reference	2.9	(0,7)	0.530	2.5	(0,7)	0.334	1.4	(0,5)	0.059
A(H3N2)	4.0	(0,10)	Reference	4.5	(0,10)	0.660	3.7	(0,11)	0.781	4.4	(0,10)	0.723
A(H1N1)pdm09	3.4	(0,8)	Reference	2.6	(0,8)	0.610	3.6	(0,10)	1.000	2.8	(0,8)	0.757
B	2.8	(0,9)	Reference	3.0	(0,11)	0.804	3.1	(0,9)	0.730	1.9	(0,9)	0.350

^#^ p-value of age group comparison using Poisson models.
