# Supplementary material for: Age-specific epidemic waves of influenza and respiratory syncytial virus in a subtropical city
Source: Sci Rep. 2015 May 18;5:10390. doi: 10.1038/srep10390 (PMC4434841; doi:10.1038/srep10390)

Supplementary Figure 1. Onset time of influenza epidemics of (A) A(H1N1), (B) A(H1N1)pdm09, (C) A(H3N2) and (D) B, in cool or warm season of each year by age groups, 2004-2013. Week number ranges from -2 to 50, with negative indicating the weeks before that corresponding year (i.e. -2 represents week 50 of last year). Warm season is defined as week 19 to week 50, and cool season as week 51 to week 18 of next year. Threshold for epidemic is set at 2% of annual total age-virus-specific positive cases.


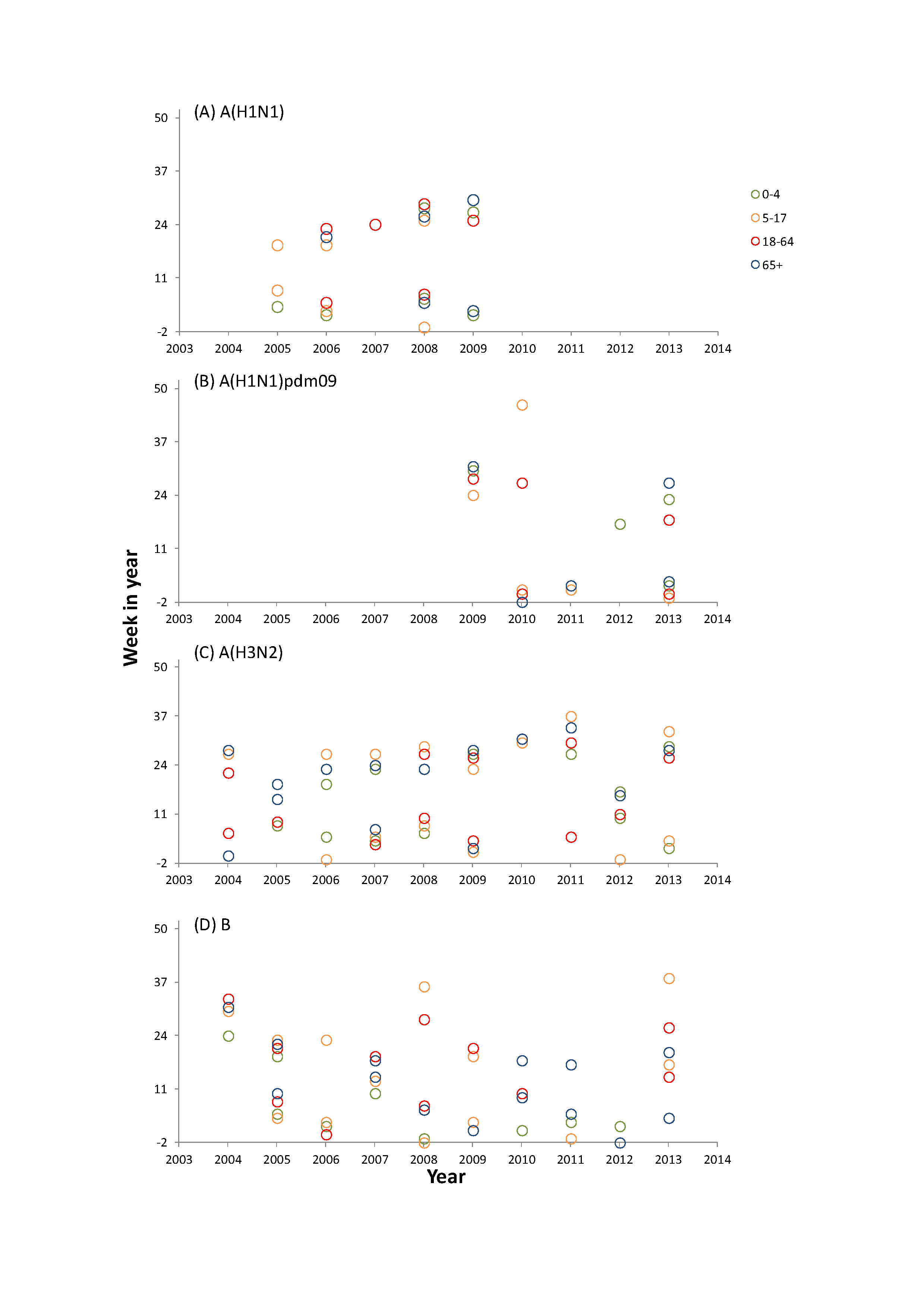

Supplement: Supplementary Information [file srep10390-s1.doc]
